# Supplementary material for: Deep learning predicts prevalent and incident Parkinson’s disease from UK Biobank fundus imaging
Source: Sci Rep. 2024 Feb 13;14:3637. doi: 10.1038/s41598-024-54251-1 (PMC10864361; doi:10.1038/s41598-024-54251-1)
Supplement: Supplementary file 1 — Supplementary Tables. [file 41598_2024_54251_MOESM1_ESM.docx]

## Supplementary

## Supplementary Table 1

The performance of the AlexNet model according to each quartile of the Parkinson’s disease diagnostic gap (years) across five randomized repetitions of five-fold cross validation. The diagnostic gap as the difference between the date of image acquisition minus the date of diagnosis, where a negative value is interpreted as having a PD diagnosis prior to fundus image acquisition (prevalent PD) and a positive value is interpreted as having a PD diagnosis post fundus image acquisition (incident PD).

| **Quartile** (diagnostic gap: years) | **N** | **Sensitivity** (mean, 95% CI) |
| --- | --- | --- |
| **Overall Parkinson’s** | 123 |  |
| Q1 (-18.72, -6.32)  Q2 (-6.32, -1.87)  Q3 (-1.87, 4.46)  Q4 (4.46, 7.38) | 31  30  30  32 | 84.52 (82.65, 85.67)  84.00 (81.71, 85.32)  76.00 (72.58, 77.97)  83.75 (81.40, 85.20) |
| **Prevalent Parkinson’s** | 77 |  |
| Q1 (-18.72, -7.63)  Q2 (-7.63, -4.80)  Q3 (-4.80, -2.04)  Q4 (-2.04, 0) | 19  19  18  21 | 81.05 (77.46, 82.76)  76.84 (74.14, 78.26)  83.33 (80.77, 84.57)  78.10 (75.00, 79.67) |
| **Incident Parkinson’s** | 46 |  |
| Q1 (0.09, 3.93)  Q2 (3.93, 5.07)  Q3 (5.07, 5.57)  Q4 (5.57, 7.38) | 11  11  12  12 | 80.00 (73.00, 82.57)  80.00 (74.11, 82.05)  93.33 (79.39, 94.83)  81.67 (79.56, 82.47) |

**Supplementary Table 2**

The performance of the AlexNet model according to male and female sub-types. Chi-squared tests of independence were used to test the significance of male and female gender on the model performance. In one instance (Incident PD-specific), a Fischer’s Exact Test as the number of instances in the contingency table was less than or equal to five.

| Dataset Category | Gender (M/F) | Metric (Male %, Female %) | p-value |
| --- | --- | --- | --- |
| Overall Parkinson’s  PD + HC  PD  HC | 142 / 106  71 / 52  71 / 52 | Accuracy (0.747 / 0.731)  Sensitivity (0.845 / 0.885)  Specificity (0.648, 0.578) | 0.781  0.530  0.424 |
| Prevalent Parkinson’s  PD + HC  PD  HC | 86 / 68  43 / 34  43 / 34 | Accuracy (0.744 / 0.721)  Sensitivity (0.721 / 0.853)  Specificity (0.767 / 0.588) | 0.434  0.166  0.688 |
| Incident Parkinson’s  PD + HC  PD  HC | 56 / 36  28 / 18  28 / 18 | Accuracy (0.768 / 0.694)  Sensitivity (0.929 / 0.848)  Specificity (0.607 / 0.667) | 0.739  0.093  0.683 |

**Supplementary Table 3**

The computation time estimates of 5-Fold-Cross-Validation for training and testing of models on color fundus images is recorded by its average and standard deviation. The number of parameters with respect to a 256 × 256 × 3 RGB image input with 2 class outputs is provided for reference and interpretation relative to the model complexity. Estimates were obtained on the University of Florida HiPerGator with 2 CPU cores and 1 NVIDIA A100 GPU.

| **Model** | Training Time (mean (std.), minutes) | Testing Time (mean (std.), seconds) | Parameters |  |  |  |
| --- | --- | --- | --- | --- | --- | --- |
| Overall (n = 246 fundus images \| 123 PD, 123 HC) | | | |  |  |  |
| Logistic Regression | 2.73 (0.02) | 0.19 (0.01) | 0.20 |  |  |  |
| Elastic Net | 14.01 (0.34) | 0.18 (0.01) | 0.20 |  |  |  |
| SVM (Linear) | 2.20 (0.03) | 1.56 (0.01) | 0.20 |  |  |  |
| SVM (RBF) | 2.75 (0.03) | 3.57 (0.17) | **0.20** |  |  |  |
| Alexnet | 19.13 (2.14) | 7.12 (2.15) | **57.0** |  |  |  |
| VGG-16 | 28.90 (1.52) | 16.14 (2.12) | 134.3 |  |  |  |
| GoogleNet | 18.03 (1.22) | 8.32 (2.43) | 12.0 |  |  |  |
| Inception-V3 | 21.52 (0.51) | 17.40 (2.12) | 21.8 |  |  |  |
| ResNet-50 | 20.32 (1.32) | 16.01 (2.50) | 23.5 |  |  |  |
| Prevalent (n = 146 fundus images \| 73 PD, 73 HC) | | | |  |  |  |
| Logistic Regression | 1.86 (0.04) | 0.11 (0.00) | 0.20 |  |  |  |
| Elastic Net | 8.45 (0.23) | 0.09 (0.01) | 0.20 |  |  |  |
| SVM (Linear) | 0.83 (0.01) | 0.63 (0.04) | 0.20 |  |  |  |
| SVM (RBF) | 1.09 (0.01) | 1.55 (0.25) | **0.20** |  |  |  |
| Alexnet | 12.20 (0.69) | 9.13 (1.97) | **57.0** |  |  |  |
| VGG-16 | 16.81 (1.54) | 15.90 (1.96) | 134.3 |  |  |  |
| GoogleNet | 11.41 (0.62) | 7.56 (2.06) | 12.0 |  |  |  |
| Inception-V3 | ­­­ 14.38 (0.96) | 16.34 (2.70) | 21.8 |  |  |  |
| ResNet-50 | 14.07 (0.95) | 14.53 (2.18) | 23.5 |  |  |  |
| Incident (n = 100 fundus images \| 50 PD, 50 HC) | | | |  |  |  |
| Logistic Regression | 1.22 (0.06) | 0.07 (0.01) | 0.20 |  |  |  |
| Elastic Net | 4.83 (0.22) | 0.08 (0.02) | 0.20 |  |  |  |
| SVM (Linear) | 0.26 (0.01) | 0.23 (0.02) | 0.20 |  |  |  |
| SVM (RBF) | 0.37 (0.01) | 0.59 (0.05) | **0.20** |  |  |  |
| Alexnet | 7.27 (0.43) | 8.48 (1.85) | **57.0** |  |  |  |
| VGG-16 | 10.13 (0.48) | 15.21 (2.50) | 134.3 |  |  |  |
| GoogleNet | 7.03 (0.18) | 7.06 (2.20) | 12.0 |  |  |  |
| Inception-V3 | 8.36 (0.63) | 16.63 (2.75) | 21.8 |  |  |  |
| ResNet-50 | 7.11 (0.31) | 14.19 (1.96) | 23.5 |  |  |  |

**Supplementary Table 4**

Gray-Scale Conversion and Vessel Segmentation Classification Results of Machine Learning Models. Values presented as mean, 95% confidence interval.

| **Model** | AUC | ACC | PPV | NPV | SENS | SPEC | F1 |
| --- | --- | --- | --- | --- | --- | --- | --- |
| Overall (n = 246 fundus images \| 123 PD, 123 HC) | | | | | | | |
| Logistic Regression (Gray) | 0.69 (0.66, 0.72) | 0.63 (0.61, 0.66) | 0.63 (0.60, 0.65) | 0.65 (0.62, 0.68) | 0.68 (0.64, 0.72) | 0.59 (0.54, 0.63) | 0.65 (0.62, 0.67) |
| Logistic Regression (Vessel) | 0.67 (0.64, 0.70) | 0.61 (0.59, 0.63) | 0.60 (0.57, 0.62) | 0.63 (0.61, 0.65) | 0.69 (0.66, 0.72) | 0.52 (0.47, 0.57) | 0.64 (0.62, 0.66) |
| Elastic Net (Gray) | 0.68 (0.64, 0.71) | 0.63 (0.59, 0.66) | 0.61 (0.58, 0.63) | 0.65 (0.61, 0.70) | 0.72 (0.68, 0.75) | 0.54 (0.49, 0.58) | 0.66 (0.63, 0.68) |
| Elastic Net (Vessel) | 0.67 (0.63, 0.70) | 0.62 (0.59, 0.64) | 0.60 (0.58, 0.62) | 0.65 (0.62, 0.68) | 0.73 (0.69, 0.77) | 0.51 (0.46, 0.56) | 0.65 (0.63, 0.67) |
| SVM (Linear, Gray) | 0.69 (0.66, 0.71) | 0.64 (0.61, 0.66) | 0.63 (0.61, 0.66) | 0.64 (0.62, 0.67) | 0.66 (0.62, 0.70) | 0.61 (0.56, 0.66) | 0.64 (0.62, 0.67) |
| SVM (Linear, Vessel) | 0.68 (0.65, 0.71) | 0.61 (0.59, 0.63) | 0.60 (0.58, 0.62) | 0.62 (0.60, 0.64) | 0.66 (0.62, 0.70) | 0.55 (0.50, 0.60) | 0.63 (0.61, 0.64) |
| SVM (RBF, Gray) | 0.73 (0.70, 0.75) | 0.70 (0.68, 0.72) | 0.67 (0.65, 0.68) | 0.76 (0.72, 0.80) | 0.80 (0.76, 0.84) | 0.59 (0.56, 0.63) | 0.72 (0.70, 0.75) |
| SVM (RBF, Vessel) | 0.65 (0.61, 0.69) | 0.61 (0.58, 0.64) | 0.56 (0.49, 0.64) | 0.64 (0.6, 0.68) | 0.65 (0.56, 0.74) | 0.57 (0.50, 0.64) | 0.60 (0.52, 0.68) |
| Prevalent (n = 146 fundus images \| 73 PD, 73 HC) | | | | | | | |
| Logistic Regression (Gray) | 0.75 (0.71, 0.78) | 0.67 (0.64, 0.71) | 0.68 (0.64, 0.72) | 0.68 (0.64, 0.72) | 0.69 (0.65, 0.73) | 0.66 (0.60, 0.72) | 0.68 (0.65, 0.71) |
| Logistic Regression (Vessel) | 0.63 (0.58, 0.67) | 0.59 (0.56, 0.63) | 0.58 (0.55, 0.61) | 0.62 (0.57, 0.66) | 0.69 (0.64, 0.73) | 0.50 (0.45, 0.54) | 0.63 (0.59, 0.66) |
| Elastic Net (Gray) | 0.72 (0.68, 0.77) | 0.68 (0.65, 0.71) | 0.66 (0.62, 0.69) | 0.72 (0.69, 0.76) | 0.76 (0.72, 0.81) | 0.59 (0.53, 0.64) | 0.70 (0.68, 0.73) |
| Elastic Net (Vessel) | 0.60 (0.56, 0.65) | 0.57 (0.53, 0.61) | 0.56 (0.52, 0.60) | 0.52 (0.43, 0.62) | 0.70 (0.63, 0.76) | 0.44 (0.35, 0.52) | 0.61 (0.58, 0.65) |
| SVM (Linear, Gray) | 0.74 (0.71, 0.78) | 0.68 (0.64, 0.71) | 0.69 (0.64, 0.73) | 0.69 (0.65, 0.72) | 0.70 (0.65, 0.74) | 0.66 (0.60, 0.73) | 0.68 (0.65, 0.72) |
| SVM (Linear, Vessel) | 0.63 (0.59, 0.67) | 0.57 (0.53, 0.61) | 0.56 (0.53, 0.60) | 0.58 (0.54, 0.62) | 0.63 (0.58, 0.68) | 0.51 (0.46, 0.56) | 0.59 (0.55, 0.63) |
| SVM (RBF, Gray) | 0.74 (0.70, 0.78) | 0.72 (0.69, 0.75) | 0.69 (0.66, 0.72) | 0.79 (0.75, 0.84) | 0.83 (0.78, 0.87) | 0.61 (0.56, 0.66) | 0.75 (0.72, 0.77) |
| SVM (RBF, Vessel) | 0.58 (0.53, 0.64) | 0.58 (0.54, 0.62) | 0.52 (0.44, 0.60) | 0.59 (0.52, 0.66) | 0.62 (0.50, 0.74) | 0.54 (0.45, 0.63) | 0.56 (0.46, 0.65) |
| Incident (n = 100 fundus images \| 50 PD, 50 HC) | | | | | | | |
| Logistic Regression (Gray) | 0.56 (0.50, 0.61) | 0.53 (0.49, 0.56) | 0.53 (0.49, 0.57) | 0.52 (0.48, 0.57) | 0.56 (0.50, 0.63) | 0.49 (0.42, 0.56) | 0.54 (0.49, 0.58) |
| Logistic Regression (Vessel) | 0.74 (0.69, 0.79) | 0.65 (0.60, 0.71) | 0.66 (0.60, 0.72) | 0.68 (0.61, 0.74) | 0.69 (0.62, 0.76) | 0.62 (0.53, 0.71) | 0.66 (0.61, 0.72) |
| Elastic Net (Gray) | 0.55 (0.51, 0.60) | 0.54 (0.51, 0.58) | 0.52 (0.46, 0.57) | 0.53 (0.44, 0.61) | 0.65 (0.55, 0.75) | 0.44 (0.34, 0.53) | 0.57 (0.50, 0.63) |
| Elastic Net (Vessel) | 0.74 (0.69, 0.78) | 0.65 (0.61, 0.70) | 0.66 (0.61, 0.71) | 0.68 (0.62, 0.74) | 0.68 (0.60, 0.75) | 0.63 (0.56, 0.71) | 0.65 (0.60, 0.70) |
| SVM (Linear, Gray) | 0.54 (0.48, 0.60) | 0.51 (0.48, 0.55) | 0.52 (0.48, 0.56) | 0.51 (0.47, 0.54) | 0.49 (0.44, 0.55) | 0.53 (0.46, 0.60) | 0.50 (0.46, 0.54) |
| SVM (Linear, Vessel) | 0.75 (0.70, 0.80) | 0.66 (0.61, 0.71) | 0.67 (0.62, 0.72) | 0.69 (0.62, 0.76) | 0.68 (0.60, 0.75) | 0.64 (0.56, 0.72) | 0.66 (0.61, 0.71) |
| SVM (RBF, Gray) | 0.66 (0.60, 0.72) | 0.63 (0.58, 0.69) | 0.58 (0.49, 0.68) | 0.65 (0.57, 0.73) | 0.65 (0.53, 0.76) | 0.62 (0.54, 0.70) | 0.60 (0.50, 0.70) |
| SVM (RBF, Vessel) | 0.56 (0.46, 0.66) | 0.57 (0.54, 0.61) | 0.40 (0.27, 0.54) | 0.58 (0.50, 0.66) | 0.43 (0.28, 0.59) | 0.72 (0.59, 0.84) | 0.40 (0.27, 0.53) |
